# Exploratory Evaluation of Peptide-Based Immunization Targeting Fusion Glycoprotein-Derived Epitopes of Nipah Virus in Murine Model

**DOI:** 10.3390/vaccines14010084

**Published:** 2026-01-13

**Authors:** Seo Young Moon, Rochelle A. Flores, Eun Bee Choi, Seungyeon Kim, Hyunjin Je, Eun Young Jang, Heeji Lim, Yoo-Kyoung Lee, In-Ohk Ouh, Woo H. Kim

**Affiliations:** 1 Division of Vaccine Development Coordination, Center for Vaccine Research, National Institute of Infectious Diseases, National Institute of Health, Korea Disease Control and Prevention Agency, Osong, Cheongju 28159, Chungcheongbuk-do, Republic of Korea; msy1477@korea.kr (S.Y.M.); dmsql2274@korea.kr (E.B.C.); hatmddus135@korea.kr (S.K.); sky11kk@korea.kr (E.Y.J.); dalgi0519@korea.kr (H.L.); leeykyoung@korea.kr (Y.-K.L.); 2 College of Veterinary Medicine, Institute of Animal Medicine, Gyeongsang National University, Jinju 52828, Gyeongsangnam-do, Republic of Korea; floresrochellea@gmail.com (R.A.F.); hjssjd29@gmail.com (H.J.)

**Keywords:** fusion protein, epitope, immunogenicity, in silico, Nipah virus, peptide vaccine, vaccine

## Abstract

**Background:** Nipah virus (NiV), a zoonotic paramyxovirus with high case fatality and pandemic potential, remains without a licensed vaccine for humans to date. Although there has been progress in vaccine development, it remains limited, and peptide vaccines have rarely been validated in vivo. **Methods:** Here, we report the rational antigen selection, synthesis, and preliminary immunogenicity evaluation of NiV fusion glycoprotein (NiV-F)-derived linear peptides as vaccine candidates. Candidate epitopes were identified by in silico, and a total of 18 B- and T-cell epitope-derived peptides were shortlisted for synthesis and antigenicity validation by ELISA. **Results:** Antigenicity evaluation showed that 9 of the synthesized peptides have A_450nm_ of over 1 (8 from the F11 group, A_450nm_: 1.13–3.6; 1 from the F18 group, A_450nm_: 1.51), with the peptide constructs F11-3 (A_450nm_: 3.5) and F11-4 (A_450nm_: 3.6) showing the highest antigenicity. Interestingly, peptides from F11 with amidation increased antibody binding (F11-4-NH2, A_450nm_: 3.05; F11-4-9mer-1-NH2, A_450nm_: 0.87). The lead peptide candidates, F11-3 and F11-4, were subsequently used for the immunization experiment, and mouse sera were assessed against their homologous peptide antigens or recombinant NiV-F protein. ELISA result showed detectable antibody reactivity against their homologous antigen for the intramuscular (IM) F11-3 vaccinated group (A_450nm_: 0.30 ± 0.35), whereas increased binding was observed for both IM-administered F11-3 (A_450nm_: 1.62 ± 0.97) and F11-4 (A_450nm_: 2.0 ± 0.77) against NiV-F protein, albeit without statistical significance compared to the negative control (NC, *p* > 0.05), and were markedly lower compared to mice immunized with NiV-F recombinant protein (PC, *p* < 0.01), underscoring the need for further optimization procedures. **Conclusions:** Collectively, these results support an exploratory antigen discovery and prioritization framework for NiV-F-derived peptide candidates and provide a foundation for future studies aimed at optimizing immunogenicity and evaluating protective relevance in appropriate preclinical models.

## 1. Introduction

Nipah virus (NiV) is a highly pathogenic zoonotic Biosafety Level 4 (BSL-4) paramyxovirus that causes sporadic but devastating encephalitis and respiratory illness, with a case fatality rate ranging less than 40% to greater than 70%, and up to 90% in some outbreaks, depending on the strain and availability of intensive support care [1,2,3,4,5]. The virus was first identified in Malaysia in 1998 and has since caused sporadic to near-annual outbreaks in South and Southeast Asia, particularly in Bangladesh and India [4,6,7]. The principal natural reservoirs of NiV are fruit bats or flying foxes of the genus *Pteropus* spp., which are widely distributed across Southeast Asia, the South Pacific, and Australia, although other hosts including other bat species and mammals such as horses and pigs have also been reported, highlighting the broad species tropism of NiV [8,9,10]. Transmission is often associated with direct bat-to-human contact, through the excretions or secretions of infected animals or consumption and exposure to food products (i.e., meat, fruits, sap, or date palm juice) contaminated by body fluids of infected animals; however, once it spreads to people, human-to-human transmission can occur [11,12]. Due to the high virulence and human-to-human transmission, NiV is classified as a priority disease requiring urgent research and development by the World Health Organization R&D Blueprint [13]. While several candidates are in development, they remain in the early stages of clinical evaluation, and the medical countermeasures available to combat NiV remains severely limited and scarce, underscoring the continued effort for effective, scalable, and innovative strategies to be developed and deployed rapidly in response to an outbreak.

NiV is an enveloped, non-segmented, single-stranded negative-sense RNA virus within the *Henipavirus* genus of *Paramyxoviridae* family. Originally, the *Henipavirus* genus included Nipah and Hendra virus (HeV), but recent discoveries now include other paramyxoviruses within the same genus including bat-borne Cedar virus (CedV), Ghanavirus (GhV), and Angavokely virus (AngV); rodent-borne Mojiang virus (MojV); and shrew-borne Gamak virus (GakV), Daeryong virus (DarV), Langya virus (LayV), Melian virus (MelV), Denwin virus (DewV), and Ninorex virus (NinExV) [14,15,16,17,18,19,20,21]. To date, only NiV, HeV, and LeV are the recognized zoonotic agents capable of spillover into human, underscoring their potential as epidemic or pandemic threats [21,22]. While the majority of the novel *henipaviruses* have been confined to their reservoir hosts, their evolutionary proximity to recognized zoonotic *henipaviruses* and the expanding geographic and host range of *henipaviruses* highlights the likelihood that additional members of this genus may also emerge with zoonotic capacity [15,16,17,19]. Together, these reinforces the importance of integrated One Health approaches, proactive surveillance, and development of cross-protective platforms as part of pandemic preparedness efforts to mitigate risks and ensure global public health security.

Within henipaviruses, NiV is the most pathogenic, transmissible, and well-characterized virus, making it an ideal prototype pathogen to elucidate viral biology and develop translational frameworks in vaccine and therapeutic strategies across relevant emerging henipaviruses and the broader *Paramyxoviridae* family. NiV is generally categorized into two major genetic lineages, sharing approximately 91.8% nucleotide homology, NiV-Malaysia (NiV-MY), and Ni-V Bangladesh (NiV-BD), each with an approximately 18.2 kilobases (kb)-long genome that encodes for six major structural proteins (surface proteins: attachment glycoprotein G (G) and fusion glycoprotein (F); matrix protein (M); viral nucleocapsid protein (N); RNA-dependent polymerase complex: phosphoprotein (P) and large protein (L)) and three accessory proteins (V, W, and C) encoded by P gene through mRNA editing or an alternative open reading frame [23,24,25]. As with other paramyxovirus, the arrangement of the genes in the NiV genome sequentially follows the order 3′-N-P-M-F-G-L-5′ [24,25]. Both NiV strains are noted to inflict pulmonary and neurological disease in humans and animal models; however, NiV-BD presents a more virulent strain associated with higher mortality rates often exceeding 70% depending on outbreak, shorter mean disease duration from onset of symptom to death at five to six days, and evidence of human-to-human transmission [6,26,27]. Similar to other paramyxoviruses, the mechanism of NiV entry to host cells is mediated by the surface proteins, attachment glycoprotein G (NiV-G) and fusion glycoprotein (NiV-F) [28]. The virus entry is initiated when the NiV-G, a tetrameric type II membrane protein, binds to the host ephrin-B2 and ephrin B3 cell receptors, which are highly conserved across vertebrates and widely expressed in the endothelial and neuronal tissues, triggering a conformational change in the NiV-G structure [29,30,31]. The conformational change in the NiV-G signals the adjacent NiV-F, a trimeric class I fusion protein, to undergo refolding, resulting in viral and cell membrane fusion and subsequent viral genome entry into the cytoplasm [32].

Together, these two surface glycoproteins, NiV-G and NiV-F, are the prime target of neutralizing antibodies, and their role in viral entry places them as the principal focus for vaccine development delivered through diverse platforms including viral vectors, recombinant protein subunits, virus-like particles (VLPs), plasmid DNA, and, more recently, mRNA technologies [28,33]. Additionally, other countermeasures of NiV infection has been investigated using small molecules, antivirals, and monoclonal antibodies [34]. However, none of these candidates, vaccine or therapeutics, has received regulatory approval for human use to date. The majority of the candidates remain in preclinical evaluation, with only one antiviral drug (ribavirin), one monoclonal antibody (mAb102.4, clinical trial identifier: ACTRN12615000395538), mRNA vaccine (mRNA-1215, NCT05398796), subunit protein (HeV-sG-V, NCT04199169), and two viral-vectored vaccines (recombinant vesicular stomatitis-vectored vaccine (PHV02), NCT05178901; recombinant adenoviral-vectored vaccine (ChadOx1 NipahB), ISRCTN87634044) in clinical trials [35,36,37,38].

Over the years, conventional vaccine platforms such as mRNA and viral-vectored vaccines have made significant epidemiological progress in controlling various diseases. However, these vaccines are often limited by persistent manufacturing and logistical constraints associated with the complex vaccine manufacturing processes, high cost of production, potential off-target immune response, and strict biosafety and cold-chain stability requirements for their global deployment [39,40,41,42]. To this end, peptide-based vaccines emerge as a compelling complementary strategy. Unlike conventional vaccine platforms, peptide-based vaccines provide precise structural and operational advantages, with the production of a chemically defined and designed focus epitopes or antigenic determinants that are favorably safe without replicative properties, thermally stable, and feasible for rapid synthesis at scale, making them suitable for timely deployment in global health emergencies or resource-limited settings where manufacturability and cross-strain protection are critical [43,44]. On the other hand, eliciting a durable protective response from peptide vaccines requires a thorough antigen selection and design, as well as strategic incorporation of potent adjuvants or optimized delivery systems, as these vaccines are also limited by their inherently low immunogenicity and high susceptibility to in vivo degradation [43,45]. Despite these challenges in peptide vaccines, recent clinical advances and successes across diverse targets, including cancer and infectious diseases such as dengue, group A streptococci, and COVID-19, demonstrate the feasibility of the platform as a safe and viable vaccine alternative to conventional vaccine strategies to elicit protective immunity [46,47,48,49,50,51,52]. However, this remains unexplored in linear NiV-derived peptide epitopes.

In the context of NiV, the fusion glycoprotein (NiV-F) is a promising vaccine target inherent to its indispensable role for viral entry and its conserved nature across NiV strains [28,32,53]. Rational epitope selection on these conserved elements of NiV-F offers avenue for a design-focused and potentially broadly protective vaccines. Building on these ideas and the evidence that F-derived peptides from heptad repeat C (HRC), especially when conjugated with cholesterol, can inhibit NiV infection in vivo by blocking membrane fusion and act as direct viral fusion inhibitors, we extend these concepts of targeting the fusion protein from therapeutic inhibition to immunogens capable of eliciting adaptive immunity to offer a potentially more durable, population-wide protection essential for pandemic preparedness [54]. Unlike previous studies that focused largely on computational immunoinformatics analyses on NiV-F, our study presents the experimental design, synthesis, and evaluation of NiV-F-derived peptides as immunogens in a murine model [55,56,57]. By synthesizing linear F-derived peptides, our work presents an exploratory antigen discovery and prioritization framework that informs the future development of epitope-driven Nipah vaccine development and extends the scope of F-targeted strategies beyond therapeutic inhibition to preventive immunization against this high consequence pathogen.

## 2. Materials and Methods

### 2.1. Study Design Overview

**Figure 1:**
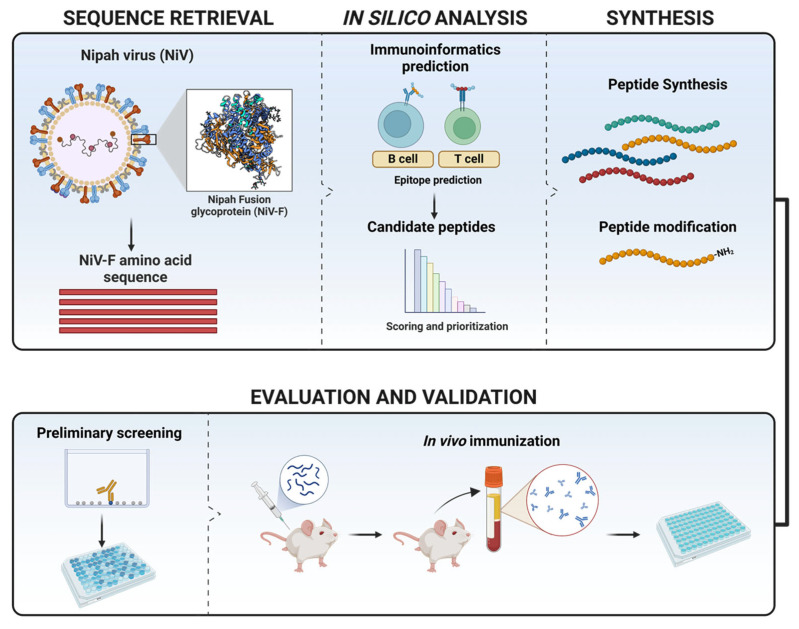
Schematic diagram of the experimental workflow for the design and evaluation of NiV-F peptide vaccine candidates. The image was generated in Biorender.com with the right to publish.

The experimental timeline of the present study followed a systematic transition starting from computational prediction to experimental validation (Figure 1). The study progressed in sequential order, following epitope prediction and antigen selection, peptide synthesis, in vitro antigenicity testing, and in vivo immunization in a murine model.

### 2.2. Epitope Prediction and Antigen Selection

The NiV-F amino acid sequence (GenBank Accession: OR947674) for vaccine design was selected based on a previously reported NiV-F consensus gene sequence, which exhibited 98.54–100% similarity across NiV strains [46]. The protein structure of NiV-F was obtained from a resolved structure in Protein Data Bank (PDB ID: 6TYS), and protein visualization was performed in a Mol*3D viewer (https://www.rcsb.org/3d-view, accessed on 1 April 2025 to 30 October 2025) [58]. Linear B-cell and T-cell epitopes of NiV-F were identified using the Immune Epitope Database and Tools (IEDB, https://www.iedb.org/, accessed on 1 January 2024 to 31 March 2024) resource as previously described [59]. Briefly, linear B-cell epitopes were predicted using BepiPred-2.0 with a 0.5 threshold and T-cell epitopes including 9-mers were screened with NetMHCpan 4.1 [60,61]. The predicted B- and T-cell epitopes were then cross-referenced, and overlapping regions with high immunogenicity scores were prioritized for peptide synthesis and downstream evaluation.

### 2.3. Peptide Synthesis

Peptides were synthesized by Fmoc solid-phase peptide synthesis (SPSS) on Rink amide resin as described [52]. In this study, selected peptides were synthesized with C-terminal amidation to assess potential effects on peptide stability and antigenicity, while the remaining peptides were produced without terminal modification. In brief, resin was pre-swollen in N, N′-dimethylforamide (DMF) for 45 min, and the amino acids (5.0 equation, Novabiochem) were coupled using 1-O-Benzotriazole-N, N,N′, N′-tetramethyl-uronium-hexafluoro-phosphate (HBTU, 5.0 equation), hydroxybenzotriazole (HOBt, 5.0 equation), and diisopropylethylamine (DIPEA, 10.0 equation) for 1 h, followed by washing and Fmoc deprotection with 20% piperidine (1 × 10 min, 2 × 3 min). This cycle of coupling, washing, and deprotection was repeated until the sequence is complete, after which resin was thoroughly washed with DMF, methanol, dichloromethane, and ether, and then vacuum-dried. Peptide were cleaved from the resin with triisopropylsilane (TIS, 5%) and H_2_O (5%) in trifluoroacetic acid (TFA, approximately 2 mL of TFA per 100 mg of resin) for 2 h, precipitated in cold ether, collected by filtration, and dried. Crude products were then analyzed and purified by reverse-phase HPLC using a 10–90% acetonitrile (0.05% TFA) over 30 min on a Phenomenex column (C18, 250 × 4.60 mm, 5 micron) or Vydac HPLC columns (C18, 250 × 10 mm, 5 micron) at 1.0 mL/min or 2.5 mL/min, respectively, with detection at 230 nm. All synthesized peptides are endotoxin-free with >95% purity.

### 2.4. Antigenicity Assay

Antigenicity preliminary screening of synthesized peptides (Table 1) were assessed by indirect ELISA using serum from mice immunized with commercial recombinant Nipah virus glycoprotein F (Human Fc-Tag, REC31633, The Native Antigen, Oxford, UK). Experimental controls included recombinant NiV-F protein as the positive control (PC) and buffer-only wells as the negative control (NC). Microplates were coated and incubated overnight with 5 µg/mL of the synthesized peptides in coating buffer following established protocols [59]. Briefly, after blocking and incubation with test serum, the plates were probed with 50 µL/well HRP-conjugated goat anti-mouse IgG (1:50,000) H + L, (Abcam, Cambridge, UK), followed by 50 µL/well 1X TMB substrate solution (Thermo Fisher Scientific, Waltham, MA, USA). The assay reaction was then subsequently stopped with stop solution (0.5 M sulfuric acid), and the absorbance was detected at absorbance 450 nm (A_450nm_) using a microplate reader (SpectraMax i3x, Molecular Devices, San Jose, CA, USA). All assays were conducted in triplicates.

### 2.5. In Vivo Validation and Evaluation

A prime–booster immunization schedule was conducted over 4 weeks in five-week-old, female, specific-pathogen-free (SPF) BALB/c mice (Samtako, Osan-si, Republic of Korea). The mice were randomly assigned and immunized twice at two-week intervals, receiving either intramuscular (IM) injections of 400 µg peptide formulated with Alhydrogel^®^ adjuvant (100 µg) or intravenous (IV) injections of 100 µg peptide (Table 2), based on a previous study [59]. Experimental controls were immunized intramuscularly with PBS (Negative Control, NC) and a positive control group (PC) receiving recombinant NiV-F protein (25 µg, Human Fc-Tag, REC31633, The Native Antigen, Oxford, UK). Antibody responses were evaluated by indirect ELISA, adapted from a previously described protocol [59]. Briefly, the plates were coated overnight at 4 °C with either their homologous peptide (5 µg/mL) or recombinant NiV-F protein to detect peptide-specific antibodies and recognition of native antigen, respectively. Microplate processing and result detection, thereafter, were performed as above in the antigenicity assay section. Mice were housed under standard laboratory conditions with ad libitum access to food and water. Daily monitoring for clinical signs including distress, lethargy, swelling on injection site, and mortality were strictly followed. Following anesthesia, blood collection was performed via the submandibular vein. All experiments involving animals were reviewed and approved by the Institutional Animal Care and Use Committee (IACUC) of the Korea Centers for Disease Control and Prevention (approval no. KDCA-IACUC-24-009, 26 March 2024).

### 2.6. Statistical Analysis

The data distribution was first assessed with the Shapiro–Wilk Test, and if at least one group did not meet normality assumptions, a non-parametric method was applied. Results are expressed as mean ± standard deviation (SD). Group comparisons were performed using the Mann–Whitney test for two-group comparison or the Kruskal–Wallis analysis with Dunn’s post hoc test for multiple comparison. Statistical significance was defined as *p*-value < 0.05. All analyses and data visualization were performed using GraphPad Prism (version 8.0, GraphPad Software Inc., San Diego, CA, USA).

## 3. Results

### 3.1. In Silico Prediction of NiV-F B- and T-Cell Epitopes

**Figure 2:**
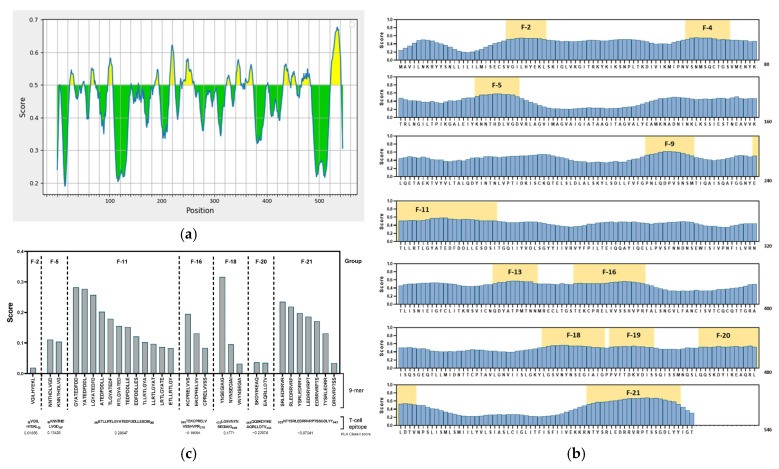
In silico prediction of B- and T-cell epitopes in of NiV-F protein sequence. (**a**) Computational analyses using BepiPred at threshold 0.5 for B-cell epitopes. Residues above the threshold and high antigenicity are indicated in yellow color. (**b**) NiV-F amino acid residue scores from BepiPred. Predicted B-cell epitope groups with over >9 amino acid residues and overlapping MHC-binding affinity are highlighted with their designated group ID. (**c**) 9-mer peptides and their HLA Class I immunogenicity scores. Vertical dashed lines indicate separation between peptide groups. F2, NiV-F epitope group 2; F4, NiV-F epitope group 4; F5, NiV-F epitope group 5; F9, NiV-F epitope group 9; F11, NiV-F epitope group 11; F13, NiV-F epitope group 13; F16, NiV-F epitope group 16; F18, NiV-F epitope group 18; F19, NiV-F epitope group 19; F20, NiV-F epitope group 20; F21, NiV-F epitope group 21.

NiV represents a major biothreat requiring the development of effective vaccine capable of eliciting broad and balanced protective immunity. Ideally, such vaccines should stimulate both humoral and cellular immune responses through the inclusion of appropriate B-cell epitopes and T-cell epitopes that can stimulate antibody production and drive helper and cytotoxic response, respectively. In this context, in silico or computational vaccinology provides a powerful and accelerated strategy for vaccine discovery by integrating specialized computational tools that can rapidly identify and prioritize epitopes exhibiting strong antigenicity and robust Major Histocompatibility Complex (MHC)-binding properties, thereby significantly reducing cost, time, and stringent biosafety requirements with traditional screening. Applying this framework in the current work, NiV-F protein was screened using BepiPred, and 21 putative linear B-cell epitopes were identified, with lengths ranging from a single residue to a 23 amino acid sequence (Figure 2a, Table S1). In order to increase the biological relevance and refine the candidate pool towards immunologically relevant motifs, a stringent in-house selection criterion of retaining only epitopes with a minimum length of ≥9 amino acids was applied. This filter reduced the set to 11 high-confidence B-cell epitope candidates distributed across distinct regions of NiV-F (Figure 2b). These B-cell epitopes were subsequently cross-referenced with T-cell binding sites to identify sequences with dual reactivity. Groups F11 (score: 0.28647), F-18 (score: 0.1771), and F5 (score: 0.13426) demonstrated the highest predicted HLA Class I affinity scores (Table S2). A parallel prediction of nonameric peptides (9-mer) further revealed multiple strong MHC binders with four candidates from F-11 (GYATEDFDD, YATEDFDDL, LGYATEDFD, ATEDFDDLL), one from F-18 (YNSEGIAIG), and two from F-21 (SRLEDRRVR, RLEDRRVRP), with a score of over 0.2 (Figure 2c, Table S3). Together, these analyses identified F-11 and F-18 as the most robust epitope region within NiV-F, supported by strong B-cell prediction scores, overlapping T-cell binding potential, and high-scoring 9-mer cores.

### 3.2. NiV-F Antigen Selection, Design, and Synthesis

**Figure 3:**
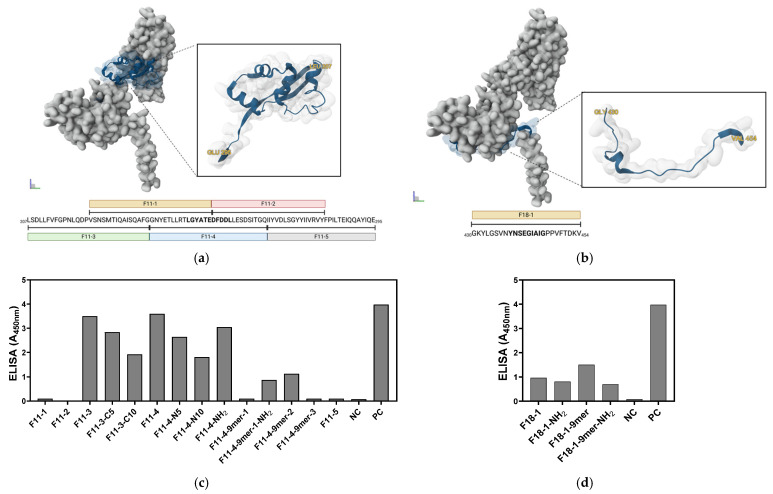
Mapping and preliminary antigenicity evaluation of the shortlisted synthesized NiV-F peptides. Location of the NiV-F epitope-derived peptide candidates, (**a**) F11 and (**b**) F18, in a resolved in monomeric NiV-F protein from PDB (6TYS). The protein was visualized in Mol*3D viewer and adjusted. The specific location of the epitope is highlighted in color and in an inlet to show the simplified cartoon representation, whereas the specific residue and subgroupings for each peptide group are shown as a sequence diagram. Indirect ELISA results of the synthesized peptide group (**c**) F11 and (**d**) F18 using sera from recombinant NiV-F vaccinated mice. Data are from triplicate values and presented as mean absorbance at 450 nm (A_450nm_).

Based on the preliminary screening above, epitopes F11 (ETLLRTLGYATEDFDDLLESDSI) and F18 (LGSVNYNSEGIAIG) were selected as core antigens for vaccine design. In order to cover a broader antigenic coverage and enhance immunogenicity of the candidates, the core sequences were elongated at either the N- or C-termini following the consolidated prediction scores from the current work and from previous prediction reports, demonstrating high immunogenicity from these residues with lengths ranging from 14 to 30 amino acids [55,57,62,63]. Similarly, the four top-ranked 9-mer peptides YNSEGIAIG (score: 0.316, F18), GYATEDFDD (0.282, F11), YATEDFDDL (0.277, F11), and LGYATEDFD (0.257, F11) were synthesized individually to assess their potential as minimal stimulatory units. F-21 9-mer was excluded due to low scores for B/T epitope overlap screening. In order to improve stability of the peptide, C-terminal amidation was introduced selectively to the top and core B/T epitopes (F11: ETLLRTLGYATEDFDDLLESDSI-NH2 and F18: LGSVNYNSEGIAIG-NH2) and top two 9-mers (F18: YNSEGIAIG-NH2 and F11: GYATEDFDD-NH2), while the remaining peptides were synthesized without modification. Collectively, the final list of NiV-F-derived epitopes for peptide synthesis were constructs from F11 and F18. The sequences, amino acid positions, and design features are summarized in Table 1, while their schematic representation and structural distribution in the NiV-F monomer structure are illustrated in Figure 3a,b. The monomeric representation is used at this stage to illustrate the location of the candidate’s peptide prior to preliminary antigenicity evaluation. Notably, mapping all residues for the F11 group were mapped into Domain III, which forms the part of the apical coiled region of NiV-F, whereas the F18 group was mapped in Domain II near the C-terminal ectodomain adjacent to the Heptad repeat region [64].

### 3.3. Antigenic Profiles of NiV-F-Derived Peptides in Mice Sera

To evaluate the antigenicity of all synthesized peptide candidates, a preliminary screening was performed using indirect ELISA with sera obtained from mice immunized with recombinant NiV-F native protein. In this assay, the synthesized individual peptides were coated onto 96-wells ELISA plates as antigens, and antibody binding from NiV-F immunized mice sera was quantified as absorbance at 450 nm (A_450nm_) values. Mean endpoint absorbance values were used in this screening to guide peptide prioritization. Nine of the fourteen synthesized peptides from the F11 group showed higher A_450nm_ values compared to NC, with the majority of the positive responses over A_450nm_ values of 1 (Figure 3c). By contrast, F18-derived peptides demonstrated a more uniform reactivity, with all the constructs higher than NC, but only one exceeded an A_450nm_ reading of 1 (F18-1-9-mer, A_450nm_: 1.51) (Figure 3d). Interestingly, terminal modification with NH2 influenced an increased antigenicity profile of the F11 peptide group (F11-4-NH2, A_450nm_: 3.05; F11-4-9mer-1-NH2, A_450nm_: 0.87) but not the F18 group (F18-1-NH2, A_450nm_: 0.81; F18-1-9-mer-NH2, A_450nm_: 0.70), as evidenced by an increased antibody binding for F11 A_450nm_ values. Based on these results, the F18 group was excluded from further testing, whereas the top F-11 constructs, which exhibited the highest antibody reactivity (F11-3, A_450nm_: 3.5; F11-4, A_450nm_: 3.6), comparable to PC (NiV-F protein, A_450nm_: 3.98), were prioritized and advanced for in vivo immunization studies.

### 3.4. Immunogenicity Evaluation of the NiV-F-Derived Peptides in Mice

**Figure 4:**
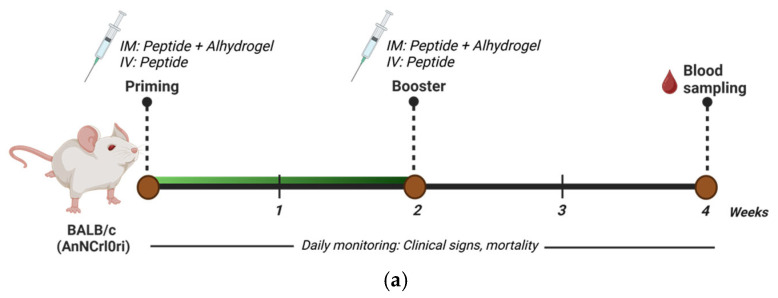

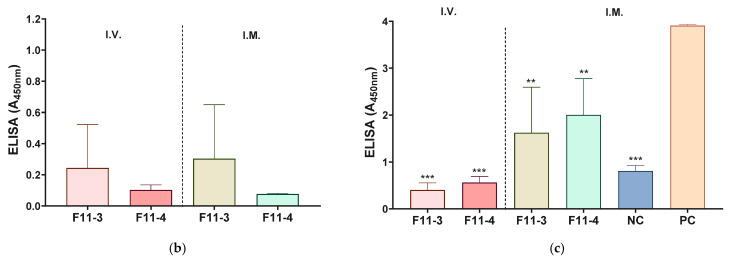
Immunogenicity evaluation of NiV-F peptide vaccine candidates in mice. (**a**) Schedule of immunization regimen and sampling collection for the in vivo validation experiment in mice. Specific-pathogen-free (SPF) mice (n = 3) were immunized twice at two-week intervals, either intramuscularly (IM. with Alhydrogel ^®^ adjuvant) or intravenously (IV). Sera were collected 2 weeks post-booster vaccination and were evaluated for humoral response by ELISA. Image was created in Biorender with license. (**b**) Antibody binding against their homologous peptide antigen. (**c**) Antibody binding against recombinant NiV-F protein. Data are presented as mean absorbance value at 450 nm (A_450nm_) ± SD. Statistical significance on all groups was determined using Kruskal–Wallis test followed by Dunn’s multiple comparison test. ***, *p* < 0.001 against positive control (PC); **, *p* < 0.01 against positive control (PC). NC, negative control.

**Figure 5:**
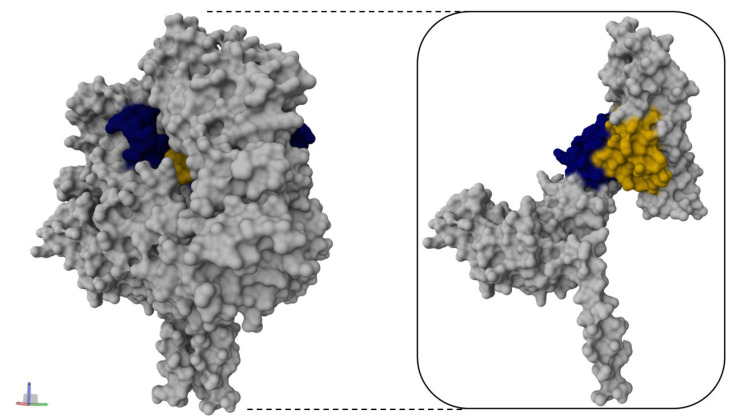
Location of the NiV-F epitope-derived F11-3 and F11-4 peptide vaccine in the trimeric NiV-F resolved protein structure. The protein structure was retrieved in PDB (6TYS), and the protein is visualized in their molecular surface representation using Mol*3D. Highlighted in blue is the specific location of F11-3, whereas yellow is for F11-4. The inlet box shows a representation of a monomeric structure of NiV-F.

Based on the preliminary screening of the peptides, F11-3 and F11-4 were subsequently evaluated in a murine immunization model as an exploratory assessment to evaluate peptide-induced antibody responses, while minimizing animal use in accordance with the 3Rs principle (Replacement, Reduction, Refinement). The vaccination regimen and experimental design are illustrated in Figure 4a. Mice were immunized either IM with Alhydrogel^®^ adjuvant or IV, and the sera were collected and evaluated using ELISA for vaccine-induced humoral responses. Specifically, to assess specificity and breath of the induced response, sera were tested against their homologous peptide to measure direct recognition of the antigen, or against recombinant NiV-F protein to evaluate cross-reactivity with the native antigen. At the end of the experiment, no other significant clinical signs or mortality were recorded in any group throughout the study period. ELISA findings showed that, when tested against their homologous peptide antigen, the IM F11-3 group exhibited the highest antibody reactivity with A_450nm_: 0.30 ± 0.35 (Figure 4b). On the other hand, in relation to the NiV-F recombinant protein, both IM-administered groups, F11-3 (A_450nm_: 1.62 ± 0.97) and F11-4 (A_450nm_: 2.0 ± 0.77), showed elevated binding relative to the NC groups (A_450nm_: 0.81 ± 0.12) but were not statistically significant (Figure 4c). In general, all IV-administered peptides showed a generally lower detectable immune response. As expected, the positive control vaccinated with the recombinant NiV-F vaccine showed the highest antibody reactivity (A_450nm_: 3.91 ± 0.02), with NC (*p* < 0.001) and all peptide groups were significantly different (IV: *p* < 0.001; IM: *p* < 0.001). The structural location of the peptides used in the in vivo trial showed their position in the central coiled domain, highlighting their spatial orientation and accessibility within the trimeric NiV-F assembly (Figure 5). Collectively, these minimally designed peptides can elicit low level, route-dependent humoral immune response that can recognize both their homologous antigen and the native protein. However, at this stage, further studies are needed to enhance their performance and immunogenicity since the magnitude of response remained significantly reduced compared with the full-length antigen immunization.

## 4. Discussion

NiV is a highly zoonotic paramyxovirus, considered a high-priority pathogen by the WHO, with broad species tropism, a high case fatality rate, and pandemic potential [13]. Since its first outbreak in 1998, no licensed human vaccine has been available, necessitating continued efforts in vaccine development as part of global preparedness efforts [5]. To date, several therapeutics and vaccine candidates are in various stages of development using different platforms [33,34]. Peptide-based vaccines, although theoretically attractive due to their ease of synthesis, high adaptability in the event of mutations during outbreaks, and generally safer nature, remain unexplored in NiV [44]. The current study investigated the design, synthesis, and evaluation of short NiV-F-derived peptides as potential immunogens in a murine model.

NiV-F is a pivotal therapeutic target in Nipah vaccine research, as it is both a major site for neutralizing antibody binding and essential for mediating the fusion of the viral and host membrane to allow viral entry to host cell [28]. In order to streamline the identification of optimal peptide vaccine candidates in this work, we employed computational analyses to determine immunologically relevant B- and T-cell epitopes of NiV-F based on their high antigenicity scores and strong predicted binding affinity to MHC molecules. Only peptides meeting a stringent threshold for both criteria were prioritized for subsequent synthesis and experimental evaluation. Similar workflows have been employed in other peptide-based vaccine studies for various infectious pathogens to increase the likelihood of identifying highly antigenic and immunogenic epitopes [65,66]. This screening and prioritization process substantially reduce the number of candidate peptides requiring synthesis and experimental validation, demonstrating a more resource-efficient pathway for early vaccine discovery, which is highly adaptable against high-containment pathogens like NiV.

Here, a total of 18 peptides were shortlisted and synthesized. The majority of these peptides were derived from the domain III region (F11 peptide group) of NiV-F, whereas the remaining are mapped to domain II (F18 peptide groups) near the C-terminal ectodomain. Preliminary antigenicity screening of the NiV-F epitope-derived peptides with serum from NiV-F recombinant protein–vaccinated mice showed that antibodies from the sera can recognize F11 peptides more strongly than those from F18. This finding can be attributed to the structural location of the F11 peptides in domain III, specifically in the apical coil region, which is more exposed and immunodominant compared to domain II, which is only transiently exposed and structurally constrained [64]. Similar patterns have been reported, where pre-fusion specific epitopes at the apex of the fusion protein elicit a stronger antibody response than the transiently exposed regions [64,67,68]. Collectively, the results validate the predictive pipeline used to identify NiV-F-derived linear peptide segments and highlight the importance of experimental validation of computational results, as well as the role of structural context in determining which linear peptide segments are most likely to reflect the naturally immunogenic region of the F protein.

Furthermore, chemical stabilization through C-terminal amidation to the top-ranked linear epitopes was investigated herein. Notably, the enhanced antibody binding in ELISA of F11-derived peptides with C-terminal amidation confirms that the modification stabilized the peptide backbone by improving the structural mimicry of the native protein, thereby promoting recognition by antibodies present in NiV-F immune sera. These results concur with the previous literature regarding the enhanced stability of the peptide and peptide–antibody recognition conferred through terminal amidation or other chemical modifications [59,69]. In contrast, amidation of F18-derived peptides yielded no comparable advantage, as their low antigenicity persisted despite the modification. The differential outcomes in the current study suggest that chemical amidation enhance the performance of surface-exposed regions but offers limited benefit for conformationally constrained or less accessible regions within the NiV-F glycoprotein structure.

Epitopes demonstrating highest antibody recognition in the preliminary ELISA-based screening were advanced to in vivo immunization studies. In this phase, selected peptides were administered to mice, and the sera were subsequently evaluated by indirect ELISA using either the homologous vaccination peptide or the recombinant NiV-F protein as coating antigens. This approach enabled qualitative evaluation of peptide-induced antibody binding and antigenic cross-recognition. In the in vivo immunization study, the strongest homologous antibody reactivity was observed in mice immunized IM with F11-3. Furthermore, when sera from these immunized mice were tested against full-length recombinant NiV-F protein, both F11-3 and F11-4 delivered via the IM route exhibited increased cross-reactive binding. The observed cross-reactivity of sera from peptide-vaccinated mice with the native glycoprotein demonstrates that these linear epitopes retain sufficient antigenic fidelity to be recognized in the context of the full fusion glycoprotein, even in the absence of a complete conformational structure [43]. By comparison, intravenous administration of the peptides consistently resulted in a low detectable immune response. This outcome is likely due to rapid clearance of the free peptides from circulation and limited engagement with antigen-presenting cells, in contrast to IM delivery with alum adjuvant, which promotes antigen depot formation and germinal center activation [44,70,71]. Aluminum salt adjuvants are the most widely used adjuvants in vaccine formulation known to elicit strong Th2 immune response [72]. In the present study, IM immunization with alum-adjuvanted peptides elicited lower antibody response compared with recombinant NiV-F protein. This difference could reflect the inherent size- and structure-related limitations, as well as the lower immunogenicity of short linear peptides relative to full-length protein antigens. Earlier reports also demonstrated limited adjuvant potency of aluminum salts for short peptide antigens [73,74]. Although aluminum adjuvants can bind to peptide antigens and modulate their release kinetics to prolong its bioavailability and enhance antigen presentation, the effectiveness of this combination is highly dependent on the charge, size, pH, hydrophobicity, and morphology of the aluminum nanoparticle [43,75]. Despite the lower magnitude of antibody responses relative to recombinant protein controls, peptide immunization elicited detectable cross-reactive antibodies against NiV-F glycoprotein, supporting the antigenic relevance of the selected linear epitopes.

## 5. Conclusions

Taken together, the results from this work present an exploratory evaluation of linear peptide epitopes derived from NiV-F Domain III, demonstrating detectable antigenicity and low-level immunogenicity following intramuscular administration in a murine model. The observed antibody recognition of recombinant NiV-F protein by sera from peptide-immunized mice indicates partial cross-reactivity with the native antigen; however, immune response was modest, variable, and did not reach statistical significance. These findings underscore the strong influence of epitope selection and route of administration on peptide immunogenicity, as well as the inherent limitations of short linear peptides relative to full-length proteins when administered with baseline aluminum-based adjuvants without structural scaffolding or optimized delivery strategies. In addition, the absence of relevant peptide controls and the high variability observed in the in vivo experiment likely reflect the small group size and the intrinsically low immunogenicity of the formulated linear peptide. Importantly, the results in the current work present an antigen discovery and prioritization effort rather than evidence of protective efficacy. To advance the translational potential of this platform, future studies should focus on strategies to enhance immunogenicity through strategies such as epitope multimerization, conjugation to inert carrier proteins, or optimized adjuvant and delivery systems. Moreover, defining functional correlates of protection using neutralization assay and cellular immune readouts, together with characterization of peptide biodistribution and in vivo persistence, will be critical to inform delivery strategies, dosing, and protective relevance in appropriate models. Collectively, these findings provide a foundation for rational refinement of peptide-based vaccines against NiV, positioning the platform as a resource-efficient and structurally defined complementary strategy within the broader landscape of NiV vaccine development.

## Figures and Tables

**Table 1:** List of Nipah virus fusion (NiV-F) glycoprotein-derived peptide sequences chemically synthesized for experimental validation.

Epitope Group	Peptide ID	Sequence	Modification	Amino Acid Position	Peptide Length (aa)
F11	F11-1	VSNSMTIQAISQAFGGNYETLLRTLGYATE		222–251	30
	F11-2	DFDDLLESDSITGQIIYVDLSGYYIIVRVY		252–281	30
	F11-3	LSDLLFVFGPNLQDPVSNSMTIQAISQAFG		207–236	30
	F11-3-C5	FVFGPNLQDPVSNSMTIQAI		212–231	20
	F11-3-C10	FVFGPNLQDPVSNSM		212–226	15
	F11-4	GNYETLLRTLGYATEDFDDLLESDSITGQ		237–265	29
	F11-4-N5	LLRTLGYATEDFDDLLESDS		242–261	20
	F11-4-N10	GYATEDFDDLLESDS		247–261	15
	F11-4-NH2	ETLLRTLGYATEDFDDLLESDSI-NH_2_	Amidation	240–261	23
	F11-4-9mer-1	GYATEDFDD		247–255	9
	F11-4-9mer-1-NH2	GYATEDFDD-NH_2_	Amidation	247–255	9
	F11-4-9mer-2	YATEDFDDL		248–256	9
	F11-4-9mer-3	LGYATEDFD		246–254	9
	F11-5	IIYVDLSGYYIIVRVY FPILTEIQQAYIQE		266–295	30
F18	F18-1	GKYLGSVNYNSEGIAIGPPVFTDKV		430–454	25
	F18-1-NH2	LGSVNYNSEGIAIG-NH_2_	Amidation	433–446	14
	F18-1-9mer	YNSEGIAIG		438–446	9
	F18-1-9mer-NH2	YNSEGIAIG-NH_2_	Amidation	438–446	9
	G17-NH_2_	NTVISRPGQSQCPRFNKC-NH_2_	Amidation	482–499	18

**Table 2:** Experimental groups and vaccination strategies for in vivo validation of Nipah virus fusion glycoprotein (NiV-F) peptide vaccine candidates.

Group	Peptide Vaccine	Route	Dose	Adjuvant *	Sample Size (n)
F11-3	F11-3 peptide	IV	100 µg/dose		3
	F11-3 peptide	IM	400 µg/dose	Alhydrogel^®^	3
F11-4	F11-4 peptide	IV	100 µg/dose		3
	F11-4 peptide	IM	400 µg/dose	Alhydrogel^®^	3
PC	NiV-F recombinant protein ^1^	IM	25 µg/dose	Alhydrogel^®^	3
NC	PBS	IM			3

IM, intramuscular; IV, intravenous; NC, negative control; PBS, phosphate-buffered saline; PC, positive control; * Alhydrogel^®^ adjuvant 2% (Invivogen, San Diego, CA, USA); ^1^ Nipah virus glycoprotein F (Human Fc-Tag; REC31633; The Native Antigen, Oxford, UK).

## Data Availability

All data used in the analyses are provided within this article and its Supplementary Materials. For any additional questions, please reach out to the authors responsible for correspondence.

## Supplementary Materials

The following supporting information can be downloaded at https://www.mdpi.com/article/10.3390/vaccines14010084/s1. Table S1: Summary of Nipah virus fusion (NiV-F) glycoprotein B-cell epitopes in silico prediction results. Table S2: Summary of the predicted T-cell epitope sequences in Nipah virus fusion (NiV-F) glycoprotein. Table S3: List of predicted Nipah virus fusion (NiV-F) glycoprotein 9-mer epitope candidates.
